# Association between clinical frailty and multidrug-resistant positive cultures in hospitalized older adults

**DOI:** 10.1007/s11739-026-04481-0

**Published:** 2026-07-29

**Authors:** Carmine Siniscalchi, Nicoletta Cerundolo, Antonella Tufano, Andrea Ticinesi, Angela Guerra, Mattia Basile, Giampiero Castaldo, Beatrice Prati, Alberto Parise, Antonio Nouvenne, Pierpaolo Di Micco, Tiziana Meschi

**Affiliations:** 1 Department of Continuity of Care and Multicomplexity, Parma University Hospital, 43126 Parma, Italy; 2 Department of Internal Medicine, Federico II University of Naples, 80100 Naples, Italy; 3 Department of Medical Sciences, University of Ferrara, Via Fossato di Mortara 64/B, 44121 Ferrara, Italy; 4 Department of Internal Medicine, “Santa Maria Delle Grazie Hospital”, Pozzuoli, Naples, Italy

**Keywords:** Multidrug-resistant bacteria, Frailty, Clinical Frailty Scale, Older adults, Antimicrobial resistance, Hospitalized patients

## Abstract

Frailty is a key determinant of adverse outcomes in older adults. The association between clinical frailty and multidrug-resistant (MDR) positive cultures in hospitalized older adults remains insufficiently defined. Understanding this association may improve early risk stratification and inform infection control strategies in acute care. We conducted a prospective, observational, single-center cohort study in an internal medicine ward. Patients aged ≥ 65 years admitted with clinical suspicion of bacterial infection requiring microbiological testing and antibiotic therapy were eligible. The diagnosis of infection was based on clinical, laboratory, and imaging findings as assessed by the treating physician, regardless of microbiological confirmation. Among 208 eligible patients with confirmed infection, participants were stratified by culture results into negative, non-MDR, and MDR + groups. Frailty was assessed using the Clinical Frailty Scale (CFS) and Primary Care Frailty Index (PC-FI), while comorbidity burden was evaluated with the Cumulative Illness Rating Scale (CIRS). Multinomial logistic regression was used to explore variables associated with MDR-positive culture status. Of the 208 patients, 57% were culture-negative, 29% had non-MDR infections and 14% MDR + . Frailty increased progressively across groups: median CFS was 5, 6, and 6 (*p* = 0.004), and median PC-FI was 0.20, 0.24, and 0.28 (*p* = 0.021). The prevalence of frailty rose from 56 to 70% to 83% (CFS > 4; *p* = 0.015). MDR + patients more frequently required full-time caregiving and had longer hospitalization and antibiotic duration. In multivariable analysis, CFS remained associated with MDR-positive culture status after adjustment for age and comorbidity burden (OR 1.57, 95% CI 1.17–2.11) although residual confounding cannot be excluded. In this selected single-center cohort of hospitalized older adults, clinical frailty was associated with MDR-positive cultures. These findings should be considered hypothesis-generating and should not be interpreted as supporting broader empirical antibiotic therapy based on frailty alone.

**Supplementary Information:**

The online version contains supplementary material available at https://doi.org/10.1007/s11739-026-04481-0.

## Introduction

Bacterial infections are a major cause of hospitalization among older adults [1], and in the presence of multimorbidity, they may rapidly progress to sepsis, contributing to functional decline, disability, and increased mortality [2, 3 ]. In this population, infections frequently present atypically, often without fever and with nonspecific symptoms such as confusion, delirium, falls, or reduced mobility, making early diagnosis challenging [4, 5 ]. At the same time, the global overuse of antibiotics has accelerated the emergence of multidrug-resistant (MDR) bacterial strains [6], defined as pathogens lacking susceptibility to at least three antimicrobial classes beyond their intrinsic resistance profile [7]. MDR organisms represent an expanding public health emergency, with projections of 10 million deaths per year by 2050 [6].

MDR organism acquisition and MDR-positive cultures occur especially in hospital settings where resistant strains are prevalent [8]. In acute geriatric and medical wards, MDR pathogens may be detected in three main circumstances: pre-existing colonization in institutionalized patients [9, 10 ], active MDR infection at admission [11], or nosocomial acquisition during the hospital stay [12]. Frailty, which is highly prevalent among acutely hospitalized older adults [13], may identify a vulnerable clinical phenotype with greater healthcare exposure, dependency, reduced physiological reserve, and possible susceptibility to infection-related complications. Frailty is characterized by reduced physiological reserve and impaired resilience to stressors [14, 15 ] and is commonly assessed using different approaches, including phenotype-based and deficit-accumulation models. In clinical practice, simplified tools based on functional autonomy in daily activities, such as the Clinical Frailty Scale (CFS), are widely used for rapid frailty assessment, while indices such as the Frailty Index (FI) are based on the deficit-accumulation model and account also for the presence of organ-specific functional impairments and illness. [16–18]. Frail individuals exhibit chronic low-grade inflammation and immune dysregulation (“inflammaging”), which may compromise the host response to infection, leading to increased vulnerability, reduced physiological reserve, slower and incomplete recovery and, ultimately, increasing the risk of adverse outcomes [19, 20 ].

Despite these plausible mechanisms, the relationship between frailty and MDR infections in hospitalized older adults remains poorly defined. Existing evidence is limited to few studies reporting high MDR prevalence in geriatric wards [21, 22 ], a post-pandemic rise in MDR infections [23], and a strong association between frailty and bacterial infections, including MDR strains, among older patients with COVID-19 [24, 25 ]. However, it remains uncertain whether frailty itself contributes to MDR-positive infections or rather reflects the cumulative effect of healthcare exposure, functional dependency, previous antibiotic use, and chronic comorbidity.

To our knowledge, no previous prospective study has specifically investigated whether different frailty instruments identify older hospitalized patients with an increased probability of MDR-positive cultures.

Therefore, the present observational study aimed to examine the association between clinical frailty and MDR-positive cultures among acutely hospitalized older adults with suspected bacterial infection. Specifically, we compared clinical characteristics, frailty levels, antibiotic use, microbiological findings, and in-hospital outcomes across culture-negative, non-MDR, and MDR-positive groups.

## Materials and methods

### Study population

This was a prospective, observational, single-center study conducted at the Internal Medicine Unit of the University Hospital of Parma, Italy, a tertiary care teaching hospital. The study was carried out between June 2024 and October 2025, enrolling all consecutive adult patients admitted to the unit with clinical suspicion of bacterial infection, based on routine assessment, laboratory, and imaging tests.

Eligible participants were aged 65 years or older and had a clinical suspicion of organ or systemic bacterial infection requiring antibiotic therapy, as determined by the treating physician on the basis of clinical, laboratory, and imaging findings. To improve diagnostic consistency and reduce potential misclassification, inclusion required the presence of at least one objective criterion supporting infection, such as elevated inflammatory markers (e.g., C-reactive protein or procalcitonin), radiological evidence of infection, or clinical signs consistent with a specific infectious focus. Nevertheless, given the complexity of diagnosing bacterial infection in older adults, particularly in the presence of atypical symptoms and frequent colonization, microbiological results were not interpreted in isolation but were evaluated together with clinical presentation, inflammatory markers, imaging findings when available, and the suspected infectious focus.

Microbiological confirmation was not required for inclusion, reflecting real-world clinical practice in older patients, in whom infections are frequently treated empirically and may remain culture-negative despite a high clinical probability of bacterial infection. This approach is consistent with previous studies conducted in acute geriatric settings [3]. We acknowledge that positive cultures, particularly from urine or screening swabs, may reflect colonization rather than true infection. However, microbiological results were not interpreted solely on the basis of culture positivity. Instead, all microbiological findings were integrated with clinical presentation, inflammatory markers, imaging findings when available, source of specimen collection, and the treating physician’s assessment to distinguish active infection from colonization. To be included in the study, participants needed to have been prescribed standard culture-based microbiological testing on body fluids upon admission, with eventual identification of the causative bacterial species and determination of its antimicrobial susceptibility profile (antibiogram). The type of microbiological sampling was not predefined by the study protocol but reflected routine clinical practice. Blood cultures, urine cultures, sputum cultures, wound swabs, rectal swabs, pharyngeal swabs, or other microbiological specimens were requested by the treating physician according to the suspected infectious source. Whenever possible, microbiological sampling was performed before initiation of antimicrobial therapy and within the first 24 h after hospital admission.

### Microbiological definitions

The classification of bacterial isolates as multidrug-resistant (MDR) followed the internationally accepted criteria proposed by Magiorakos et al. and subsequently endorsed in global surveillance analyses by the Antimicrobial Resistance Collaborators, defining MDR organisms as non-susceptible to at least one agent in three or more antimicrobial categories. [6, 7 ]. Isolates obtained from surveillance cultures (e.g., rectal or pharyngeal swabs) were considered as colonization unless supported by clinical, laboratory, or imaging evidence of active infection. Patients with colonization only were not classified in the MDR-positive group. To improve the reproducibility of infection classification, the site-specific adjudication process used to distinguish active infection from colonization is summarized in Supplementary Fig. S2. The adjudication algorithm was predefined before data analysis and applied consistently to all enrolled patients.

Delirium was assessed based on clinical evaluation by the treating physician according to standard diagnostic criteria (e.g., DSM-5), as part of routine clinical practice.

### Infection adjudication and colonization assessment

To reduce misclassification between active infection and colonization, all positive microbiological results were reviewed in relation to the suspected infectious focus, clinical presentation, inflammatory markers, imaging findings when available, and antibiotic treatment decisions. Urinary tract infection was considered likely only in the presence of compatible urinary symptoms and/or systemic signs of infection together with urine culture positivity. Positive urine cultures in the absence of compatible clinical findings were interpreted cautiously as possible colonization or asymptomatic bacteriuria. Rectal and pharyngeal swabs were considered markers of colonization unless there was supporting clinical, laboratory, or imaging evidence of active infection attributable to the same organism. However, microbiological results were not interpreted solely on the basis of culture positivity. Instead, all microbiological findings were integrated with clinical presentation, inflammatory markers, imaging findings (when available), source of specimen collection, and the treating physician’s assessment to distinguish active infection from colonization. Cases with uncertain distinction between colonization and infection were reviewed individually and were not used to support clinically actionable conclusions. When uncertainty persisted after clinical review, cases were conservatively classified as non-definitive infection and were not considered as evidence supporting clinically actionable conclusions regarding MDR infection.

### Data collection

Clinical and laboratory data were collected prospectively using a standardized electronic database, named “RedCap”. For each patient, demographic data, comorbidities, infection severity, vital signs, laboratory parameters (including inflammatory markers), and microbiological results were collected. To ensure accurate classification, cases with MDR isolates were individually reviewed to distinguish true infection from colonization. Frailty was assessed using validated instruments, including the Clinical Frailty Scale (CFS) and the Primary Care Frailty Index (PC-FI), while comorbidity burden was evaluated using the Cumulative Illness Rating Scale (CIRS). The CFS was intended to reflect the patient’s premorbid baseline functional status rather than the acute severity of the infectious episode. Whenever possible, the score was assigned using information obtained from the patient, caregivers, previous clinical records, and functional status before the acute hospitalization. These tools capture complementary aspects of patient vulnerability, although they do not replace a comprehensive geriatric assessment. Specifically, the CFS primarily reflects functional autonomy and mobility through clinician assessment of activities of daily living and instrumental activities of daily living, whereas the PC-FI is based on the accumulation of predefined health deficits, including chronic diseases, organ dysfunction, and functional impairment. Microbiological data encompassed pathogen identification, antimicrobial susceptibility profiles, and MDR classification. Treatment variables included empirical and targeted antimicrobial regimens, use of combination therapy, and treatment duration. Outcomes of interest comprised length of hospital stay and in-hospital mortality. Patients and/or the public were not involved in the design, conduct, reporting, or dissemination of this research.

### Statistical analysis

Continuous variables were expressed as median and interquartile range, while categorical variables were presented as number and percentages. Kruskal–Wallis test or Pearson’s Chi-square were used for comparisons between patients with culture-negative, non-MDR and MDR +, while *p* for trend calculated with Jonckheere–Terpstra or Mantel–Haenszel. For blood culture comparisons in non-MDR and MDR + patients, Mann–Whitney test was used for continuous variables and Yates’ Chi-squared test or Fisher’s exact test for categorical variables. Separate multinomial and binary logistic regression analyses were performed to explore associations between clinical variables and microbiological outcomes. The analyses were interpreted as exploratory and hypothesis-generating. The term “associated” was preferred over “predicted” because the observational design does not allow causal inference or direct clinical decision rules for empirical antibiotic escalation. Variables significantly associated in univariate analyses were subsequently entered into stepwise regression models. Given the limited number of events, especially when comparing patients with positive and negative blood cultures, the models were kept parsimonious to reduce the risk of overfitting. Because of this, the regression model may be unstable and prone to overestimation of effect sizes. Established MDR risk factors available in the dataset, including recent hospitalization, residence in nursing home, need for full-time professional caregiving, and other proxies of healthcare exposure, were considered when clinically appropriate. However, some important variables, including previous antibiotic exposure, prior MDR colonization or infection, chronic urinary catheterization, invasive devices, and previous broad-spectrum antibiotic therapy, were not systematically available and were therefore addressed as potential unmeasured confounders. Because several established MDR determinants were unavailable, the objective of the regression analysis was not to establish causality but to explore whether frailty remained associated with MDR-positive cultures after adjustment for the principal confounders available in the dataset.

Statistical analyses were performed using IBM SPSS Statistics version 29.0 (IBM Corp., Armonk, NY, USA), and all tests were two-tailed with a significance threshold of *p* < 0.05.

The study protocol was approved by the Local Ethics Committee of the University Hospital of Parma (protocol number: 216/2024/OSS/AOUPR, date of approval 18th June 2024) and conducted in accordance with the principles of the Declaration of Helsinki. Written informed consent was obtained from all participants prior to inclusion in the study.

## Results

A total of 208 consecutive patients with clinical suspicion of bacterial infection who underwent microbiological testing upon admission were enrolled. After comprehensive clinical evaluation integrating laboratory, imaging, microbiological findings, and treating physician assessment, all were considered to have bacterial infection and were included in the final analysis according to the predefined adjudication criteria described in the Methods. They were stratified into three groups according to the outcomes of microbiological tests: 119 (57%) with negative cultures, 60 (29%) with positive cultures and isolation of non-multidrug-resistant (non-MDR) bacteria, and 29 (14%) with positive cultures and isolation of multidrug-resistant (MDR +) bacteria. The median age was 83 years (IQR 78–88), and 51% were male, with no significant differences between groups. Most patients were living at home (over 90%) and were assisted by family members or caregivers, with a higher proportion of full-time professional care among MDR + patients (31% vs 11% in other groups; *p* = 0.027) (Table 1). Clinical presentation was often non-specific. Fever was present in 39% of negative, 37% of non-MDR, and 28% of MDR + patients. Dysuria was more frequent among MDR + cases (7%, *p* = 0.027). Neurological manifestations were common in older adults, with confusion reported in 10–15%, and delirium in up to 5%, without intergroup differences (Table 1). Urinary tract infections represented the most frequent site of infection across the cohort. Positive urine cultures were obtained in 90% of patients with MDR infections, compared with 73% of those with non-MDR infections. Although the overall comparison among the three study groups (negative, non-MDR, and MDR +) reached statistical significance (*p* < 0.001), the direct comparison between the MDR + and non-MDR groups showed only a trend toward significance (*p* = 0.080), suggesting a higher, but not statistically confirmed, prevalence of urinary tract involvement among patients with MDR bacteria. Positive blood cultures occurred in 28% of MDR + and 22% of non-MDR patients (*p* = 0.543 MDR + vs non -MDR). Percentages reported for microbiological specimens refer to the proportion of patients within each study group who had at least one positive culture from the specified site. Sputum cultures were positive in 3% of MDR + patients, while rectal and pharyngeal swabs were positive in 7% and 3%, respectively. (Table 1).

**Table 1:** Clinical presentation and microbiological testing in patients (n.208) stratified according to microbiological results: culture-negative-no positive cultures; non-multidrug-resistant (MDR), at least one culture positive for non-MDR bacteria only; and MDR +, at least one culture positive for MDR bacteria, with or without non-MDR isolates

Parameter	Culture-negative (*N *= 119, 57%)	Non-MDR (*N* = 60, 29%)	MDR + (N.29, 14%)	*p*	*p* for trend*
Age, years	83 (77–88)	85 (79–90)	84 (79–87)	0.332	
Males, (*N*) %	(59) 50	(31) 52	(16) 55	0.857	
Weight, kg	72 (61–82)	73 (56–81)	64 (55–73)	0.328	
Place of living of patients					
In nursing home, (*N*) %	(10) 8	(4) 7	(2) 7	0.915	
At his own home, (*N*) %	(108) 91	(54) 92	(27) 93	0.920	
* Assisted by husband/wife, (N) %*	(48) 48	(24) 45	(9) 35	0.498	
* No, alone, (N) %*	(20) 20	(16) 30	(6) 23	0.350	
* Yes, by his son/daughter, (N) %*	(22) 22	(6) 11	(3) 12	0.187	
* Yes, by a professional caregiver h24, (N) %*	(11) 11	(6) 11	(8) 31	**0.027**	**0.029**
Clinical presentation					
Fever, (*N*) %	(46) 39	(22) 37	(8) 28	0.540	
Shiver, (*N*) %	(2) 2	(5) 8	(0) 0	**0.037**	
Dyspnea, (*N*) %	(41) 34	(16) 27	(10) 34	0.552	
Cough, (*N*) %	(13) 11	(2) 3	(2) 7	0.208	
Stranguria, (*N*) %	(7) 6	(8) 13	(1) 3	0.137	
Dysuria, (*N*) %	(1) 1	0 (0)	(2) 7	**0.027**	
Erythema, (*N*) %	(5) 4	(1) 2	(1) 3	0.674	
Swelling indicative of local infections, (*N*) %	(47) 39	(25) 42	(13) 45	0.862	
Confusion, (*N*) %	(14) 12	(9) 15	(3) 10	0.769	
Delirium, (*N*) %	(6) 5	(3) 5	(0) 0	0.467	
Recent fall or worsening balance, (*N*) %	(19) 16	(14) 23	(6) 21	0.471	
Rapid deterioration of cognitive function, (*N*) %	(18) 15	(7) 12	(1) 3	0.228	
Reduction of appetite, (*N*) %	(18) 15	(7) 12	(2) 7	0.466	
None**,** (*N*) %	(43) 36	(14) 23	(8) 28	0.197	
Empirical treatment, (*N*) %	(1) 1	(5) 8	(6) 21	** < 0.001**	** < 0.001**
Worsening of clinical conditions, (*N*) %	(2) 2	(0) 0	(1) 3	0.418	
Microbiological testing					
Positive blood culture, (*N*) %	(0) 0	(13) 22	(8) 28	** < 0.001**	** < 0.001**
Positive urine culture, (*N*) %	(0) 0	(44) 73	(26) 90	** < 0.001**	** < 0.001**
Positive sputum culture, (*N*) %	(0) 0	(1) 2	(1) 3	0.187	
Positive fecal culture, (*N*) %	(0) 0	(3) 5	(0) 0	**0.023**	
Positive skin swab, (*N*) %	(0) 0	(0) 0	(0) 0	/	
Positive pharyngeal swab, (*N*) %	(0) 0	(1) 2	(1) 3	0.187	
Positive rectal swab, (*N*) %	(0) 0	(0) 0	(2) 7	**0.045**	
Multidrug-resistant pathogens isolated					
ESBL Enterobacteriaceae, (*N*) %	(0) 0	0 (0)	(12) 41	** < 0.001**	** < 0.001**
KPC *Klebsiella pneumoniae*, (*N*) %	(0) 0	(0) 0	(7) 24	** < 0.001**	** < 0.001**
* Clostridium difficile* or Enterococcus resistant to vancomycin, (*N*) %	(0) 0	(0) 0	(0) 0	**/**	**/**
Other, (*N*) %	(0) 0	(0) 0	(8) 28	** < 0.001**	** < 0.001**
Antimicrobial susceptibility testing					
Resistance to penicillins first generation (amoxicillin), (*N*) %	(0) 0	(0) 0	(26) 90	** < 0.001**	** < 0.001**
Resistance to penicillins second generation (piperacillin), (*N*) %	(0) 0	(0) 0	(19) 68	** < 0.001**	** < 0.001**
Resistance to carbapenems, (*N*) %	(0) 0	(0) 0	(10) 36	** < 0.001**	** < 0.001**
Resistance to cephalosporins first or second generation (cefixime), (*N*) %	(0) 0	(0) 0	(11) 39	** < 0.001**	** < 0.001**
Resistance to cephalosporins third generation (ceftriaxone), (*N*) %	(0) 0	(0) 0	(12) 43	** < 0.001**	** < 0.001**
Resistance to fluoroquinolones, (*N*) %	(0) 0	(0) 0	(6) 21	** < 0.001**	** < 0.001**
Resistance to macrolides, (*N*) %	(0) 0	(0) 0	(8) 29	** < 0.001**	** < 0.001**
Resistance to vancomycin or teicoplanin, (*N*) %	(0) 0	(0) 0	(3) 11	** < 0.001**	** < 0.001**
Resistance to daptomycin, (*N*) %	(0) 0	(0) 0	(7) 25	** < 0.001**	** < 0.001**
Resistance to aminoglycosides,(*N*) %	(0) 0	(0) 0	(2) 7	**0.002**	
Resistance to trimetoprim/sulfometoxazole, (*N*) %	(0) 0	(0) 0	(10) 36	** < 0.001**	** < 0.001**
Resistance to Other, (*N*) %	(0) 0	0 (0)	(1) 4	**0.040**	

*MDR*   multidrug-resistant, *ESBL*  extended-spectrum beta-lactamase, *KPC*   Klebsiella pneumoniae carbapenemase, *IQR*   interquartile range

Data are reported as median (IQR) or number (percentage), with percentages calculated within each group. *p* calculated with Kruskal–Wallis or Pearson’s Chi-square, p for trend calculated with Jonckheere–Terpstra or Mantel–Haenszel

*P* values < 0.05 are indicated in bold

*P* trend* values are not reported if not significant

Importantly, isolates interpreted as colonization alone were excluded from the MDR-positive group according to the predefined adjudication algorithm (Supplementary Fig. S2). Therefore, all patients included in the MDR-positive group fulfilled the predefined clinical criteria for active infection (Table 2).

**Table 2:** Comorbidities, CIRS scale, Clinical Frailty Scale (CFS), and Primary Care Frailty Index (PC-FI) in patients stratified according to microbiological results

Parameter	Culture-negative (*N* = 119, 57%)	Non-MDR (*N* = 60, 29%)	MDR + (N.29, 14%)	*p*	*p* for trend*
Comorbidities					
Severe disability, (*N*) %	(42) 35	(22) 37	(16) 55	0.135	
Cardiovascular disease, (*N*) %	(45) 38	(20) 33	(13) 45	0.573	
Solid neoplasm, (*N*) %	(28) 24	(18) 30	(10) 34	0.401	
COPD, (*N*) %	(44) 37	(12) 20	(10) 34	0.066	
Ischemic heart disease, (*N*) %	(37) 31	(17) 28	(12) 41	0.452	
Heart failure, (*N*) %	(42) 35	(21) 35	(10) 34	0.996	
Chronic kidney disease, (*N*) %	(37) 31	(18) 30	(10) 34	0.911	
Arrhythmia, (*N*) %	(30) 26	(20) 33	(9) 31	0.560	
Atrial fibrillation, (*N*) %	(22) 18	(14) 23	(9) 31	0.315	
Diabetes, (*N*) %	(33) 29	(21) 35	(9) 31	0.693	
Obesity, (*N*) %	(30) 26	(18) 30	(12) 41	0.283	
Dyslipidemia, (*N*) %	(51) 44	(30) 50	(18) 62	0.208	
Hypertension, (*N*) %	(75) 64	(37) 62	(21) 72	0.594	
Cirrhosis, (*N*) %	(4) 3	(3) 5	(0) 0	0.475	
Parkinsonism, (*N*) %	(9) 8	(1) 2	(0) 0	0.094	**0.037**
Previous hip fracture, (*N*) %	(16) 13	(3) 5	(1) 3	0.093	**0.040**
Anemia, (*N*) %	(39) 33	(27) 45	(13) 45	0.202	
Chronic gastro, (*N*) %	(6) 5	(4) 7	(2) 7	0.886	
Dementia, (*N*) %	(11) 9	(6) 10	(6) 21	0.211	
Previous stroke, (*N*) %	(10) 9	(3) 5	(0) 0	0.219	
Partial or total financial support for medical expenses, (*N*) %	(29) 24	(13) 22	(6) 21	0.873	
Oxygen prescription in the last 6 months, (*N*) %	(20) 17	(9) 15	(10) 34	0.062	
Any hospitalization in the previous 6 months, (*N*) %	(33) 28	(15) 25	(13) 45	0.132	
Chronic ulcers of the skin, (*N*) %	(13) 11	(4) 7	(4) 14	0.521	
Bradycardia/rhythm conduction disorders, (*N*) %	(14) 12	(4) 7	(6) 21	0.151	
Neurological diseases other than dementia or Parkinson, (*N*) %	(15) 13	(8) 13	(6) 21	0.523	
Constipation, (*N*) %	(44) 37	(22) 37	(13) 45	0.715	
Prescription of enoxaparin fondaparinux in the last 6 months, (*N*) %	(34) 29	(16) 27	(13) 45	0.179	
Peripheral vascular disease, (*N*) %	(16) 13	(12) 20	(10) 34	**0.029**	**0.010**
Nutritional problems/malnutrition, (*N*) %	(26) 22	(16) 27	(8) 28	0.691	
Schizophrenia or other delusional disease, (*N*) %	(6) 5	(1) 2	(2) 7	0.441	
Edema, (*N*) %	(8) 7	(0) 0	(5) 17	**0.007**	
Cumulative Illness Rating Scale					
CIRS-CARD	2 (0–3)	2 (0–3)	2 (1–3)	0.564	
CIRS-HYPER	2 (2–2)	2 (2–2)	2 (2–2)	0.616	
CIRS-RESP	2 (0–3)	1 (0–3)	1 (0–2)	0.689	
CIRS-VASC	0 (0–1)	1 (0–2)	2 (0–2)	**0.009**	**0.002**
CIRS-ORL	0 (0–0)	0 (0–1)	0 (0–2)	**0.002**	** < 0.001**
CIRS-upper gastrointestinal	0 (0–1)	0 (0–1)	0 (0–2)	0.238	
CIRS-lower gastrointestinal	0 (0–1)	0 (0–2)	0 (0–2)	0.093	**0.029**
CIRS -LIVER	0 (0–1)	0 (0–1)	0 (0–2)	0.105	**0.036**
CIRS-KIDNEY	0 (0–2)	1 (0–2)	2 (0–3)	0.607	
CIRS-URO	0 (0–2)	1 (0–2)	2 (0–2)	**0.036**	**0.009**
CIRS-MUSC	1 (0–2)	1 (0–2)	2 (0–3)	0.096	
CIRS-NERV	0 (0–1)	1 (0–2)	1 (0–2)	0.255	
CIRS-ENDO	1 (0–3)	1 (0–2)	1 (0–2)	0.609	
CIRS-PSY	0 (0–1)	1 (0–2)	0 (0–2)	**0.026**	**0.010**
CIRS-CS	11 (6–16)	13 (7–21)	12 (8–27)	0.096	**0.029**
CIRS-SI	2 (1–4)	2 (1–3)	3 (1–5)	**0.041**	
CFS	5 (4–6)	6 (4–7)	6 (5–7)	**0.004**	**0.002**
CFS > 4, (*N*) %	(67) 56	(42) 70	(24) 83	**0.015**	**0.004**
PC-FI	0.20 (0.12–0.32)	0.24 (0.12–0.32)	0.28 (0.24–0.44)	**0.021**	**0.045**
PC-FI > 0.20, (*N*) %	(55) 47	(34) 57	(23) 79	**0.006**	**0.002**

*COPD*   chronic obstructive pulmonary disease, *CIRS*   cumulative illness rating scale, *CFS*   clinical frailty scale, *PC-FI*  primary care frailty index, *IQR*   interquartile range

Data are reported as median and IQR or number and percentage. *p* calculated with Kruskal–Wallis or Pearson’s Chi-square, p for trend calculated with Jonckheere–Terpstra or Mantel–Haenszel

*P* values < 0.05 are indicated in bold. *p trend-values are not reported if not significant

Among MDR isolates, the most frequent pathogens were ESBL-producing Enterobacteriaceae (41%), carbapenem-resistant *Klebsiella pneumoniae* (24%), and other resistant Gram-negative organisms (28%). No vancomycin-resistant enterococci or *Clostridioides difficile*-resistant strains were identified. Antimicrobial susceptibility testing demonstrated high resistance rates to first-generation penicillins (90%), second-generation penicillins (68%), cephalosporins (up to 43% for third generation), fluoroquinolones (21%), macrolides (29%), carbapenems (36%), and trimethoprim–sulfamethoxazole (36%) (Table 1). The median duration of antibiotic therapy was 10 days (IQR 7–15) in MDR + infections, compared with 7 days (IQR 5–10) in other groups. (*p* = 0.012Table 3). Hospital stay length mirrored MDR status, being 14 days (IQR 9–20) in MDR + patients, significantly longer than in non-MDR (8 days, IQR 6–13) or negative (8 days, IQR 6–12) cases (*p* < 0.001*, Table 3). Discharge to long-term or rehabilitation facilities occurred in 4% of MDR + versus 0% and 1% of other patients, while mortality during hospitalization remained relatively low (4% in MDR +, 2% in non-MDR, and 2% in negative patients; *p* = 0.795) (Table 3).

**Table 3:** Laboratory findings and outcomes stratified by microbiological results

Parameter	Culture-negative (*N* = 119, 57%)	Non-MDR (*N* = 60, 29%)	MDR + (N.29, 14%)	*p*	*p* for trend*
Hematology and clinical chemistry					
Hemoglobin, g/dl	11.3 (9.8–13.1)	11.8 (10.3–13.0)	10.5 (9.4–11.9)	**0.044**	
White blood cells, n/mm^3^	8490 (6480–12060)	8855(6298–11835)	9645 (5798–11,258)	0.995	
Neutrophils, n/mm^3^	6530 (4313–9525)	7043 (4228–9325)	7298 (4930–9835)	0.919	
Lymphocytes, n/mm^3^	995 (670–1473)	1097 (766–1554)	893 (569–1440)	0.552	
Platelets, 1000/mm^3^	197 (149–260)	198 (149–259)	228 (179–332)	0.265	
Sodium, mEq/L	139 (137–141)	139 (136–141)	140 (138–142)	0.690	
Potassium, mEq/L	3.9 (3.6–4.3)	3.9 (3.6–4.2)	3.9 (3.6–4.2)	0.611	
Urea, mg/dl	55 (34–79)	50 (37–73)	54 (37–75)	0.902	
Creatinine, mg/dl	1.1 (0.8–1.4)	0.9 (0.7–1.3)	1.0 (0.8–1.7)	0.584	
Total bilirubin, mg/dl	0.7 (0.6–1.0)	0.7 (0.5–1.2)	0.6 (0.5–0.8)	0.311	
AST, UI/L	26 (20–40)	23 (17–40)	30 (24–37)	0.192	
LDH, UI/L	206 (169–277)	193 (155–260)	201 (164–266)	0.419	
CPK, UI/L	88 (45–162)	71 (31–157)	101 (37–236)	0.493	
Baseline CRP, mg/L	66 (21–135)	94 (22–178)	84 (20–151)	0.493	
Baseline PCT, ng/ml	0.32 (0.13–1.04)	0.63 (0.09–6.18)	0.40 (0.10–3.63)	0.246	
White blood cell peak	9495 (7278–12,658)	9405 (6798–12,595)	11,110 (6780–14045)	0.666	
Creatinine peak	1.1 (0.8–1.6)	1.0 (0.7–1.4)	1.0 (0.9–1.7)	0.532	
CRP peak	77 (27–151)	100 (32–181)	92 (35–151)	0.577	
PCT peak	0.32 (0.12–1.06)	0.56 (0.09–4.24)	0.71 (0.13–8.16)	0.234	
Baseline CRP < 5 mg/L, (*N*) %	(9) 8	(3) 5	(2) 7	0.796	
Baseline CRP ≥ 5 and < 40 mg/L, (*N*) %	(30) 28	(13) 23	(9) 32	0.674	
Baseline CRP ≥ 40 and < 200 mg/L, (*N*) %	(60) 55	(31) 55	(13) 46	0.696	
Baseline CRP ≥ 200 mg/L, (*N*) %	(10) 9	(9)16	(4) 14	0.400	
CRP peak < 5 mg/L, (*N*) %	(8) 7	(4) 7	(1) 4	0.791	
CRP peak ≥ 5 and < 40 mg/L, (*N*) %	(29) 26	(12) 20	(6) 21	0.692	
CRP peak ≥ 40 and < 200 mg/L, (*N*) %	(64) 57	(33) 56	(17) 61	0.915	
CRP peaks ≥ 200 mg/L, (*N*) %	(11) 10	(10) 17	(4) 14	0.395	
Baseline PCT < 0.05 ng/ml, (*N*) %	(4) 4	(1) 2	(2) 8	0.530	
Baseline PCT ≥ 0.05 ng/ml and < 0.5 ng/ml, (*N*) %	(55) 56	(17) 40	(12) 48	0.185	
Baseline PCT ≥ 0.5 ng/ml and < 2 ng/ml, (*N*) %	(22) 22	(10) 23	(3) 12	0.479	
Baseline PCT ≥ 2 ng/ml, (*N*) %	(17) 17	(15) 35	(8) 32	**0.049**	**0.036**
PCT peak < 0.05 ng/ml, (*N*) %	(4) 4	(2) 4	(2) 8	0.716	
PCT peak ≥ 0.05 ng/ml and < 0.5 ng/ml, (*N*) %	(54) 54	(21) 43	(9) 35	0.130	**0.045**
PCT peak ≥ 0.5 ng/ml and < 2 ng/ml, (*N*) %	(21) 21	(9) 18	(5) 19	0.914	
PCT peak ≥ 2 ng/ml, (*N*) %	(20) 20	(17) 35	(10) 38	0.063	**0.025**
Antibiotic duration, days	7 (5–10)	7 (5–10)	10 (7–15)	**0.012**	**0.036**
Length of stay, days	8 (6–12)	8 (6–13)	14 (9–20)	** < 0.001**	** < 0.001**
Outcomes					
Discharge to a long-term care or rehabilitation setting, (*N*) %	(1) 1	(0) 0	(1) 4	0.274	
Transferred to another ward, (*N*) %	(2) 2	(0) 0	(1) 4	0.405	
Discharge to higher intensity of care setting, (*N*) %	(5) 4	(4) 7	(3) 11	0.391	
Discharge to the previous care setting, (*N*) %	(109) 92	(55) 92	(22) 79	0.104	
Second infection, (*N*) %	(3) 3	(1) 2	(2) 7	0.361	
Functional decline, (*N*) %	(23) 19	(12) 23	(10) 36	0.167	
Delirium, (*N*) %	(8) 7	(7) 12	(3) 10	0.569	
Hospital mortality, (*N*) %	(2) 2	(1) 2	(1) 4	0.795	

*AST* aspartate aminotransferase, *LDH*  lactate dehydrogenase, *CPK*   creatine phosphokinase, *CRP *C-reactive protein, *PCT*  procalcitonin, *IQR* interquartile range

Data are reported as median and IQR or number and percentage

*p* calculated with Kruskal–Wallis or Pearson’s Chi-square, *p* for trend calculated with Jonckheere–Terpstra or Mantel–Haenszel

*P* values < 0.05 are indicated in bold. *p trend values are not reported if not significant

**Fig. 1:**
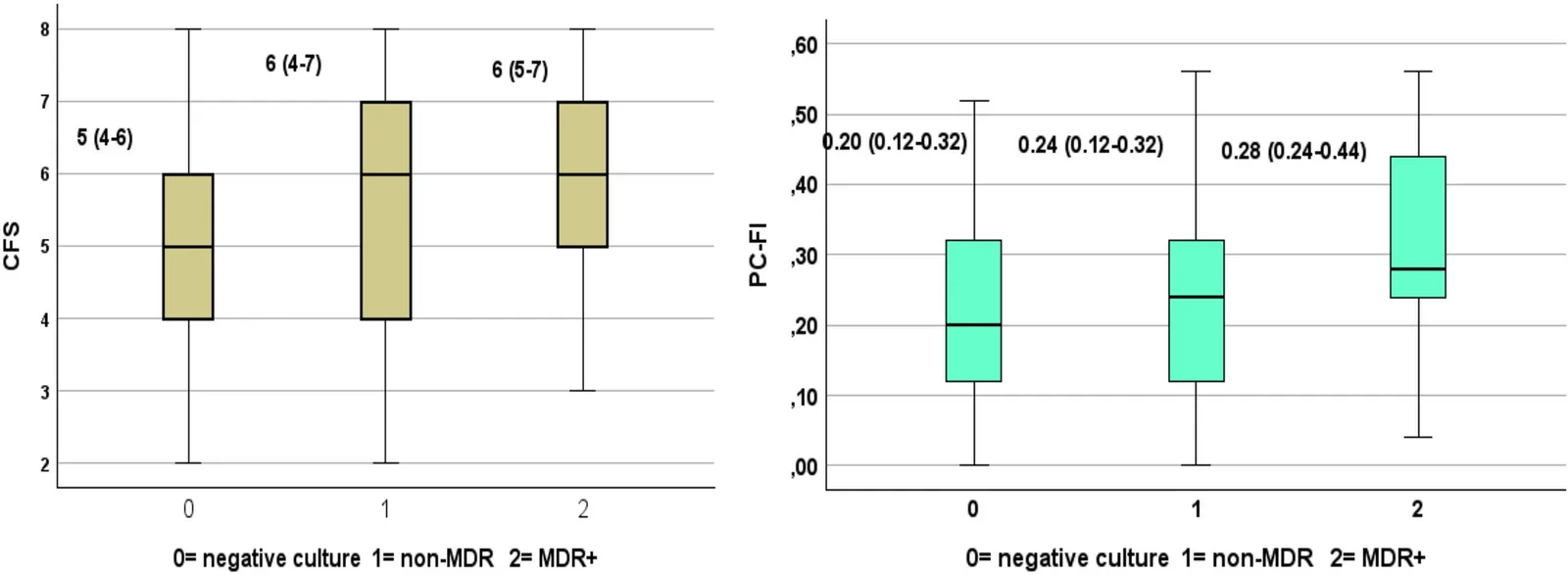
Clinical Frailty Scale and Frailty Index in patients stratified by negative, non-multidrug-resistant, and multidrug-resistant results

Frailty assessments showed higher functional vulnerability among patients with MDR-positive cultures. CFS, PC-FI, and selected CIRS domains were higher among MDR-positive patients compared with culture-negative and non-MDR groups (Fig. 1 and Table 2). Although both frailty instruments showed progressive increases across microbiological groups, only CFS remained associated with MDR-positive culture status after multivariable adjustment. When these variables were entered into a stepwise multinomial logistic regression, the frailty score (CFS) remained associated with MDR-positive culture status (*p* = 0.003; OR 1.57, 95% CI 1.17–2.11*) (Table 4). Nevertheless, because several established MDR risk factors were unavailable for adjustment, this association should be interpreted as exploratory rather than demonstrating an independent causal relationship.

**Table 4:** Multinomial logistic regression, stepwise method, variables associated with patients stratified into negative, non-multidrug-resistant, and multidrug-resistant

		*p*	Odds ratio	95,0% CI for Odds ratio
Patients non-MDR vs patients with culture negative	CFS	0.083	1.193	0.978–1.455
Patients MDR + vs patients with culture negative	CFS	**0.003**	1.572	1.172–2.109
Patients MDR + vs patients non-MDR +	CFS	0.084	1.318	0.964–1.802

Age and CIRS severity index were forced into the multinomial regression model as predefined confounders, while variables significantly associated in univariate analyses were entered using a stepwise approach. Among the tested variables, only CFS retained statistical significance in the final model

*MDR*  multidrug-resistant, *CFS*  clinical frailty scale, *CIRS*   cumulative illness rating scale, *CI*   confidence interval, *OR*   odds ratio

Bold font indicates statistically significant results (*P* 0.05)

In patients with positive blood cultures (*n* = 21), 14 (67%) also showed a concomitant urinary infection. Among MDR + patients with bacteremia (*n* = 8), two also harbored non-MDR isolates, but MDR pathogens were consistently detected in urine samples. Clinical severity markers, including confusion, rapid deterioration of cognitive function, and elevated procalcitonin levels, were significantly more frequent in patients with positive blood cultures (*p* < 0.05) (Table 5). Biochemical parameters reflected systemic inflammation: median CRP peak was 153 mg/L (IQR 64–226) and 188 (118–227) in non-MDR and MDR + infections, respectively, and peak procalcitonin was 7.39 ng/mL (IQR 2.84–44.2) and 19.89 (2.38–75.87) in non-MDR and MDR + infections. Elevated baseline PCT ≥ 2 ng/mL was observed in 67% and 86% of non-MDR and MDR + patients with positive blood culture vs. 26% and 11% of the respective patients with negative blood culture. Logistic regression showed that PCT peak ≥ 2 ng/mL (OR 20.949, 95% CI 3.267–134.333, *p* = 0.001), lymphocyte ≤ 600/mm^3^ (OR 16.786, 95% CI 2.639–106.785, 0.003), and confusion (OR 27.731, 95% CI 2.225–345.659, *p* = 0.010) were independently associated with positive blood cultures (Table 6). However, the very large odds ratio observed, for the variables present in this regression together with the extremely wide confidence interval, indicates a high degree of model instability, likely due to the limited number of bloodstream infection events. Therefore, this finding should be interpreted with caution and considered hypothesis-generating rather than definitive. These analyses were not intended to validate MDR infection classification, but rather to describe clinical and laboratory features associated with bloodstream involvement in this selected cohort.

**Table 5:** Patient stratification by multidrug resistance status and blood culture results

	Blood culture in patients non-MDR			Blood culture in patients MDR +		
	Positive N. 13	Negative N.47	p	Positive N. 8	Negative N.21	*p*
Age, years	87 (78–90)	85 (79–90)	0.864	81 (75–84)	84 (81–87)	0.093
Males, (*N*) %	(6) 46	(25) 53	0.892	(4) 50	(12) 57	1.000
CFS	6 (5–7)	6 (3–7)	0.164	7 (6–7)	6 (5–7)	0.093
CFS > 4, (*N*) %	(10) 77	(32) 68	0.736	(8) 100	(16) 76	0.283
PC-FI	0.24 (0.10–0.34)	0.24 (0.12–0.32)	0.545	0.30 (0.25–0.41)	0.24 (0.22–0.44)	0.756
PC-FI > 0.20, (*N*) %	(9) 69	(25) 53	0.358	(7) 88	(16) 76	0.647
Fever, (*N*) %	(5) 38	(17) 36	1.000	(5) 63	(3) 14	**0.019**
Dysuria, (*N*) %	(0) 0	(0) 0	/	(2) 25	(0) 0	0.069
Confusion, (*N*) %	(5) 38	(4) 9	**0.018**	(2) 25	(1) 5	0.176
Rapid deterioration of cognitive function, (*N*) %	(4) 31	(3) 6	**0.034**	(1) 13	(0) 0	0.276
Bradycardia/rhythm conduction disorders, (*N*) %	(3) 23	(1) 2	**0.029**	(1) 13	(5) 24	0.647
Dementia, (*N*) %	(3) 23	(3) 7	0.114	(3) 38	(3) 14	0.305
White Blood Cell, n/mm^3^	9905 (6010–15358)	8785(6298–11,533)	0.485	5700 (4080–10410)	10,060 (7440–11620)	0.089
Neutrophils, n/mm^3^	7407 (4639–13,792)	7043 (4197–9036)	0.391	5043 (3458–8571)	7907 (5184–10,116)	0.182
Lymphocytes, n/mm^3^	793 (318–1133)	1139 (838–1662)	**0.022**	529 (301–952)	1002 (798–1705)	**0.011**
Lymphocytes, ≤ 600/mm^3^, (N) %	(5) 38	(4) 9	**0016**	(5) 62	(3) 15	**0.022**
Platelets, 1000/mm^3^	161 (142–186)	219 (157–273)	**0.022**	190 (129–272)	235 (179–347)	0.429
Creatinine, mg/dl	1.0 (0.7–1.4)	0.9 (0.7–1.4)	0.765	0.9 (0.7–1.0)	1.1 (0.8–2.1)	0.168
Baseline CRP, mg/L	83 (50–181)	94 (22–178)	0.725	188 (118–227)	36 (17–97)	**0.002**
Baseline PCT, ng/ml	9.6 (0.69–44.5)	0.49 (0.08–2.48)	**0.004**	19.89 (2.38–75.87)	0.20 (0.06–0.50)	** < 0.001**
White blood cell peak, n/mm^3^	12,470 (7285–15,551)	9185 (6855–12,423)	0.111	5700 (4113–10,925)	12,245 (9005–14780)	**0.028**
Creatinine peak, mg/dl	1.1 (0.8–1.4)	0.9 (0.7–1.4)	0.570	0.9 (0.7–1.0)	1.2 (0.9–2.25)	0.083
CRP peak, mg/L	153 (64–226)	94 (22–169)	0.116	188 (118–227)	73 (24–113)	**0.001**
PCT peak, ng/ml	7.39 (2.84–44.2)	0.41 (0.07–1.89)	** < 0.001**	19.89 (2.38–75.87)	0.42 (0.09–0.89)	**0.001**
Baseline CRP ≥ 200 mg/L, (*N*) %	(2) 16	(7) 16	1.000	(3) 43	(1) 5	**0.038**
CRP peak ≥ 200 mg/L, (*N*) %	(3) 25	(7) 15	0.409	(3) 43	(1) 5	**0.038**
Baseline PCT < 0.05 ng/ml, (*N*) %	(0) 0	(1) 3	1.000	(0) 0	(2) 11	1.000
Baseline PCT ≥ 0.05 ng/ml and < 0.5 ng/ml, (*N*) %	(1) 11	(16) 47	0.065	(0) 0	(12) 67	**0.005**
Baseline PCT ≥ 0.5 ng/ml and < 2 ng/ml, (*N*) %	(2) 22	(8) 24	1.000	(1) 14	(2) 11	1.000
Baseline PCT ≥ 2 ng/ml, (*N*) %	(6) 67	(9) 26	**0.046**	(6) 86	(2) 11	**0.001**
PCT peak < 0.05 ng/ml, (N) %	(0) 0	(2) 5	1.000	(0) 0	(2) 11	0.750
PCT peak ≥ 0.05 ng/ml and < 0.5 ng/ml, (*N*) %	(1) 10	(20) 51	**0.030**	(0) 0	(9) 47	0.058
PCT peak ≥ 0.5 ng/ml and < 2 ng/ml, (*N*) %	(1) 10	(8) 21	0.663	(1) 14	(4) 21	1.000
PCT peak ≥ 2 ng/ml, (*N*) %	(8) 80	(9) 23	**0.002**	(6) 86	(4) 21	**0.005**
Hospital mortality, (*N*) %	(0) 0	(1) 2	1.000	(0) 0	(1) 5	1.000
Second_infection, (*N*) %	(0) 0	(1) 2	1.000	(1) 13	(1) 5	0.483
Functional decline,(*N*) %	(5) 38	(9) 19	0.251	(6) 86	(4) 19	**0.003**
Delirium, (*N*) %	(2) 15	(5) 11	0.757	(3) 38	(0) 0	**0.015**

*MDR*   multidrug-resistant, *CFS*   clinical frailty scale, *PC-FI*   primary care frailty index, *CRP*  C-reactive protein, *PCT*  procalcitonin, *IQR * interquartile range

Data are reported as median and interquartile range or percentages. *p* calculated with Mann–Whitney test or Yates’ Chi-squared test or Fisher’s exact test, as appropriate

*P* values < 0.05 are indicated in bold

**Table 6:** Variables associated with positive blood cultures in binary logistic regression analysis, stepwise method, in patients with positive culture (N.89) without stratification for non-multidrug-resistant, and multidrug-resistant

	*p*	Odds Ratio	95,0% CI per odds ratio
Confusion (present vs absent)	**0.010**	27.731	2.225–345.659
Lymphocytes ≤ 600/mm^3^	**0.003**	16.786	2.639–106.785
PCT peak ≥ 2 ng/mL	**0.001**	20.949	3.267–134.333

R^2^ di Nagelkerke = 0.595

*OR*  odds ratio; *CI *  confidence interval; *PCT*   procalcitonin; *MDR*  multidrug-resistant

Variables tested in the regression: confusion, lymphocytes (n/mm^3^, 100/mm^3^, ≤ 600/mm^3^), PCT (peak ≥ 2, ng/ml)

Bold font indicates statistically significant results (*P* 0.05)

## Discussion

In this prospective, monocentric study of 208 older adults admitted to an Internal Medicine Unit, we found that MDR-positive cultures were relatively frequent, approximately one in seven patients in the overall cohort, although this estimate is influenced by the high proportion of culture-negative cases and should therefore be interpreted with caution. Surveillance isolates were carefully evaluated, and only those associated with clinical evidence of infection were included in the MDR-positive group. Some MDR isolates were obtained from surveillance swabs (e.g., rectal swabs), which may overestimate the burden of true infection. To improve methodological transparency, we predefined site-specific adjudication criteria before patient classification and summarized the complete algorithm in Supplementary Fig. S2. Although this approach cannot completely eliminate misclassification, the predefined adjudication process substantially improved the reproducibility of infection classification and reduced the risk of misclassifying colonization as active infection compared with classification based on microbiological positivity alone. Although overall in-hospital mortality remained low, MDR-positive status was associated with a significantly longer hospital stay, higher frailty burden, and greater clinical and laboratory severity, as reflected by elevated procalcitonin and C-reactive protein levels. These findings suggest a clinically relevant association between frailty, antimicrobial resistance, and healthcare burden in older inpatients, but they should not be interpreted as evidence that frailty alone identifies patients requiring broader empirical antibiotic therapy.

The predominance of urinary and bloodstream infections in this cohort mirrors patterns observed in previous studies of older hospitalized populations, in which urosepsis represents a leading cause of bacteremia and antibiotic exposure [26]. Notably, MDR pathogens, especially carbapenem-resistant *Klebsiella pneumoniae* and extended-spectrum beta-lactamase (ESBL)-producing Enterobacteriaceae, accounted for more than two-thirds of resistant isolates. This distribution is consistent with the European Centre for Disease Prevention and Control (ECDC) surveillance data, which continue to show the predominance of Enterobacterales and *Pseudomonas aeruginosa* among nosocomial MDR pathogens in Southern Europe. [27].

A key finding of this study is the association between higher CFS scores and MDR-positive cultures. This association should not be interpreted as evidence that frailty per se causes MDR infection or as a criterion for broader empirical antibiotic therapy. Rather, CFS may capture a complex clinical phenotype characterized by reduced functional reserve, dependency, recurrent healthcare exposure, and vulnerability to adverse infectious outcomes. Importantly, PC-FI did not retain statistical significance in the multivariable model, suggesting that the relationship between frailty and MDR status may depend on the specific dimension of frailty captured by each instrument. Therefore, our findings should be considered hypothesis-generating and require confirmation in larger cohorts with more detailed assessment of established MDR risk factors.

The discordance between CFS and PC-FI deserves particular consideration because these instruments capture complementary but distinct dimensions of frailty. This observation may also explain why only the CFS remained associated with MDR-positive cultures after adjustment, whereas the PC-FI did not. Although both instruments quantify frailty, they assess different dimensions of vulnerability. The PC-FI is primarily based on the accumulation of predefined health deficits, chronic diseases, and organ-specific dysfunction, whereas the CFS incorporates clinician-assessed functional reserve, mobility, dependence, and overall biological vulnerability. Therefore, the persistence of an association for CFS but not for PC-FI suggests that functional impairment and reduced physiological reserve, rather than multimorbidity alone, may better characterize the clinical phenotype associated with MDR-positive cultures. This hypothesis, however, requires confirmation in larger multicenter studies specifically designed to compare different frailty instruments.

An alternative explanation deserves consideration. Clinical frailty may not directly increase biological susceptibility to MDR organisms but rather summarize multidimensional vulnerability that is difficult to capture individually, including recurrent healthcare exposure, dependence on caregivers, previous hospitalizations, chronic medical complexity, and cumulative antibiotic pressure. In this context, the CFS may function as an integrative clinical marker rather than a direct biological determinant of antimicrobial resistance. Future studies specifically adjusting for previous antibiotic exposure, prior MDR colonization, urinary catheterization, invasive devices, and cumulative healthcare exposure will be necessary to clarify whether frailty contributes to MDR-positive infections or primarily reflects these established determinants.

From an antimicrobial stewardship perspective, our findings should not be interpreted as supporting empirical antibiotic escalation based solely on frailty. Instead, frailty assessment should be regarded as one component of a comprehensive clinical evaluation integrating microbiological findings, previous healthcare exposure, prior antibiotic history, source of infection, and current guideline recommendations before empirical antimicrobial therapy is selected. Frailty may therefore assist clinicians in identifying patients requiring closer microbiological assessment and early reassessment of empirical therapy, rather than broader empirical antimicrobial coverage.

In our cohort, higher PCT levels were associated with bloodstream infection. Because this analysis was exploratory and not designed to evaluate diagnostic biomarkers, these findings should not be interpreted as supporting the use of PCT for diagnosing bacterial infection or guiding empirical antimicrobial therapy. Rather, elevated PCT likely reflects greater systemic inflammatory burden and disease severity in patients with bloodstream infection and should not be considered sufficient to distinguish active MDR infection from colonization [28]. Our findings align with recent global estimates from the Antimicrobial Resistance Collaborators, who reported that bacterial antimicrobial resistance was directly responsible for more than 1.27 million deaths worldwide in 2019, with the greatest burden occurring among older adults and in regions characterized by high levels of antibiotic consumption [29]. In this context, the modest mortality observed in our cohort (4% among MDR + patients) may reflect early identification and multidisciplinary management. Nonetheless, the marked prolongation of hospital stay and frequent use of broad-spectrum antibiotics highlight the substantial healthcare burden associated with MDR infections, even when clinical outcomes appear favorable.

Our findings suggest that patients with MDR-positive cultures represent a subgroup characterized by increased clinical complexity and greater healthcare resource utilization rather than by increased short-term mortality. Longer hospital stays, prolonged antibiotic treatment, and higher frailty burden indicate that these patients require multidisciplinary management and careful antimicrobial stewardship. Future studies should investigate whether frailty can contribute to identifying patients requiring enhanced microbiological surveillance without promoting unnecessary broad-spectrum antibiotic use.

The present study has several limitations. It was conducted in a single center, which may limit the generalizability of findings to other healthcare environments. Although data collection was prospective, the sample size of MDR-positive cases was modest, potentially underpowering some subgroup analyses. Although hospitalization during the previous 6 months was available, detailed information regarding the number of admissions, cumulative healthcare contacts, and emergency department visits was not systematically collected and therefore could not be incorporated into the regression model. Finally, long-term outcomes beyond hospital discharge were not available, and mortality data reflect only the in-hospital period. An additional limitation is the potential misclassification of infection, as a substantial proportion of patients had culture-negative results. In older adults, the diagnosis of infection is often based on clinical judgment, and microbiological confirmation may be lacking despite a high clinical suspicion. Because microbiological confirmation was not mandatory for inclusion, some patients in the culture-negative group may have had non-bacterial inflammatory conditions despite the predefined clinical criteria adopted to support the diagnosis of infection. Moreover, positive cultures, particularly from urine samples, may reflect colonization rather than true infection. Moreover, the study was not specifically designed to evaluate the independent contribution of individual MDR risk factors, and therefore the regression analyses should be interpreted as exploratory. This issue is particularly relevant in older adults, in whom asymptomatic bacteriuria is common and urinary cultures may be difficult to interpret without clear urinary symptoms or systemic evidence attributable to the urinary tract. Furthermore, several well-established determinants of MDR infection, including previous antibiotic exposure, previous MDR colonization or infection, chronic urinary catheterization, invasive devices, and cumulative healthcare exposure, were not systematically available and therefore could not be fully incorporated into the multivariable models. Consequently, residual confounding is likely, and the observed association between CFS and MDR-positive cultures should not be interpreted as evidence of a direct causal relationship. Finally, the relatively small number of MDR cases and bloodstream infections may have contributed to instability in multivariable models, as reflected by wide confidence intervals for some estimates. Consequently, residual confounding cannot be excluded, and the observed association between CFS and MDR-positive cultures should be interpreted as hypothesis-generating rather than causal.

Despite these precautions, some degree of misclassification between colonization and infection cannot be entirely excluded. Although we attempted to minimize this limitation through predefined adjudication criteria and standardized clinical review (Supplementary Fig. S2), complete elimination of classification bias is not possible in observational studies involving frail hospitalized older adults. However, our findings provide valuable insight into the epidemiology and clinical implications of MDR infections in older adults hospitalized in internal medicine wards. The combination of frailty assessment, microbiological surveillance, and biomarker profiling offers a multidimensional framework for risk stratification. From a clinical standpoint, integrating frailty evaluation into antimicrobial stewardship programs could help prioritize early de-escalation, infection control measures, and discharge planning. From a public health perspective, these data reinforce the need for continued surveillance and for the development of therapeutic strategies addressing the intersection between aging, chronic comorbidity, and antimicrobial resistance.

## Conclusion

In this prospective single-center cohort of hospitalized older adults with suspected bacterial infection, higher clinical frailty was associated with the presence of MDR-positive cultures. However, because of the observational design, small number of MDR cases, potential misclassification between infection and colonization, and residual confounding by established MDR risk factors, these findings should be considered hypothesis-generating. Frailty assessment should not be used alone to justify broader empirical antibiotic therapy, but may contribute to a multidimensional evaluation including clinical presentation, microbiological data, prior healthcare exposure, and antimicrobial stewardship principles. Whether frailty represents an independent biological determinant of MDR-positive infection or a surrogate marker of cumulative healthcare exposure requires confirmation in larger multicenter studies specifically designed to address this question. Future multicenter studies incorporating detailed microbiological adjudication and established MDR risk factors are required before frailty assessment can be considered for integration into clinical decision-making regarding MDR-positive infections.

## Supplementary Information

Below is the link to the electronic supplementary material.

Supplementary file1 (DOCX 1440 KB)

## Data Availability

Data are available on reasonable request. Deidentified participant data coded for analysis are available from the corresponding author. Data are available for further analysis based on the agreement of all coauthors.
