# Supplementary material for: Association between clinical frailty and multidrug-resistant positive cultures in hospitalized older adults
Source: Intern Emerg Med. 2026 Jul 29;21(8):2873–86. doi: 10.1007/s11739-026-04481-0 (PMC13751574; doi:10.1007/s11739-026-04481-0)
Supplement: Supplementary file 1 [file 11739_2026_4481_MOESM1_ESM.docx]

**Supplementary Table S1**. Chronic medications in patients (n.208) stratified according to microbiological results.

| Parameter | **Culture-negative**  (N=119, 57%) | **non-MDR**  (N=60, 29%) | **MDR+**  (N.29, 14%) | p | p for trend* |
| --- | --- | --- | --- | --- | --- |
| Chronic medications, number | 6 (4-9) | 7 (4-9) | 7 (5-10) | 0.345 |  |
| ACE inhibitors, (N) % | (24) 20 | (18) 31 | (7) 24 | 0.281 |  |
| ARBs, (N) % | (27) 23 | (17) 29 | (5) 17 | 0.451 |  |
| Calcium antagonists, (N) % | (16) 14 | (10) 17 | (7) 25 | 0.349 |  |
| Beta blockers, (N) % | (56) 47 | (23) 38 | (14) 48 | 0.496 |  |
| Alpha_blockers, (N) % | (11) 9 | (6) 10 | (5)17 | 0.456 |  |
| Vasodiatators, (N) % | (0) 0 | (2) 3 | (0) 0 | 0.082 |  |
| Diuretics, (N) % | (44) 37 | (18) 31 | (11) 38 | 0.643 |  |
| Thiazides, (N) % | (17) 14 | (7) 12 | (3) 10 | 0.799 |  |
| Statins, (N) % | (53) 45 | (35) 59 | (15) 52 | 0.174 |  |
| Steroids, (N) % | (12) 10 | (5) 8 | (2) 7 | 0.830 |  |
| Antiplatelet drugs, % | (54) 45 | (27) 45 | (13) 45 | 0.998 |  |
| OAC, (N) % | (6) 5 | (7) 12 | (1) 3 | 0.178 |  |
| NOAC, (N) % | (25) 21 | (10) 17 | (9) 31 | 0.331 |  |
| Bronchodilators, (N) % | (5) 4 | (2) 3 | (0) 0 | 0.525 |  |
| Antidepressant, (N) % | (15) 13 | (8) 14 | (5) 17 | 0.816 |  |
| Typical antipsychotics, (N) % | (2) 2 | (4) 7 | (3) 10 | 0.071 | **0.022** |
| Atypical antipsychoticsatyp, (N) % | (10) 8 | (4) 7 | (4) 14 | 0.528 |  |
| Antiepileptics, (N) % | (9) 8 | (5) 8 | (1) 3 | 0.678 |  |
| Paracetamol, (N) % | (6) 5 | (2) 3 | (0) 0 | 0.435 |  |
| Opioid, (N) % | (10) 8 | (7) 12 | (3) 10 | 0.775 |  |
| PPI, (N)% | (69) 58 | (32) 53 | (16) 55 | 0.833 |  |
| Prokinetics, (N)% | (2) 2 | (1) 2 | (0) 0 | 0.780 |  |
| Insulin, (N) % | (16) 13 | (10) 17 | (5) 17 | 0.790 |  |
| Hypoglycemic, (N) % | (19) 16 | (12) 20 | (4) 14 | 0.710 |  |
| Supplementary nutrition, (N) % | (21) 18 | (17) 29 | (3) 10 | 0.085 |  |

Data reported as median and IQR or number and percentage. p calculated with Kruskal-Wallis or chi square, p for trend calculated with Jonckheere Terpstra or Mantel Haenszel. P values <0.05 are indicated in bold

**Supplementary Table S2.** Clinical criteria used to distinguish active bacterial infection from colonization according to the site of microbiological isolation.

| **Site** | **Criteria considered compatible with active infection** | **Criteria suggestive of colonization** |
| --- | --- | --- |
| Urinary tract | Urinary symptoms ± systemic signs + positive urine culture | Positive urine culture without compatible urinary/systemic findings |
| Bloodstream | Positive blood cultures judged clinically significant | Contaminants |
| Respiratory | New infiltrate + respiratory symptoms + culture | Colonization of upper airways |
| Skin/Soft tissue | Local inflammatory signs + positive culture | Surface contamination |
| Rectal swab | Never considered infection alone | Colonization |
| Pharyngeal swab | Never considered infection alone | Colonization |

Site-specific clinical adjudication algorithm used in the study to classify positive microbiological findings as active bacterial infection or colonization. Classification was based on the integration of microbiological results with clinical presentation, inflammatory markers, imaging findings (when available), and the presumed site of infection. Rectal and pharyngeal swabs were considered evidence of colonization unless supported by additional clinical evidence of active infection.

**Supplementary Figure S2.** Site-Specific Clinical Adjudication Algorithm for the Classification of Active Infection and Colonization

**MDR**, multidrug-resistant; **EUCAST**, European Committee on Antimicrobial Susceptibility Testing; **CFU**, colony-forming units; **UTI**, urinary tract infection; **CRP**, C-reactive protein; **PCT**, procalcitonin; **WBC**, white blood cell count.

**Supplementary Figure S2.** Flowchart illustrating the predefined adjudication process used to classify microbiological isolates as active infection or colonization. Microbiological findings were interpreted by integrating the specimen source with the patient's clinical presentation, laboratory markers, imaging findings (when available), and physician assessment. Positive surveillance cultures (e.g., rectal or pharyngeal swabs) were considered indicative of colonization unless supported by compatible clinical evidence of active infection. Only patients fulfilling the predefined criteria for active infection were included in the MDR-positive or non-MDR infection groups.
